# Mild Traumatic Brain Injury (mTBI) and chronic cognitive impairment: A scoping review

**DOI:** 10.1371/journal.pone.0174847

**Published:** 2017-04-11

**Authors:** Kerry McInnes, Christopher L. Friesen, Diane E. MacKenzie, David A. Westwood, Shaun G. Boe

**Affiliations:** 1 Laboratory for Brain Recovery and Function, Dalhousie University, Halifax, Nova Scotia, Canada; 2 School of Physiotherapy, Dalhousie University, Halifax, Nova Scotia, Canada; 3 Department of Psychology and Neuroscience, Dalhousie University, Halifax, Nova Scotia, Canada; 4 School of Occupational Therapy, Dalhousie University, Halifax, Nova Scotia, Canada; 5 School of Health and Human Performance, Dalhousie University, Halifax, Nova Scotia, Canada; University of Florida, UNITED STATES

## Abstract

Mild traumatic brain injury (mTBI), or concussion, is the most common type of traumatic brain injury. With mTBI comes symptoms that include headaches, fatigue, depression, anxiety and irritability, as well as impaired cognitive function. Symptom resolution is thought to occur within 3 months post-injury, with the exception of a small percentage of individuals who are said to experience persistent post-concussion syndrome. The number of individuals who experience persistent symptoms appears to be low despite clear evidence of longer-term pathophysiological changes resulting from mTBI. In light of the incongruency between these longer-term changes in brain pathology and the number of individuals with longer-term mTBI-related symptoms, particularly impaired cognitive function, we performed a scoping review of the literature that behaviourally assessed short- and long-term cognitive function in individuals with a single mTBI, with the goal of identifying the impact of a single concussion on cognitive function in the chronic stage post-injury. CINAHL, Embase, and Medline/Ovid were searched July 2015 for studies related to concussion and cognitive impairment. Data relating to the presence/absence of cognitive impairment were extracted from 45 studies meeting our inclusion criteria. Results indicate that, in contrast to the prevailing view that most symptoms of concussion are resolved within 3 months post-injury, approximately half of individuals with a single mTBI demonstrate long-term cognitive impairment. Study limitations notwithstanding, these findings highlight the need to carefully examine the long-term implications of a single mTBI.

## Introduction

Mild traumatic brain injury (mTBI), more commonly known as concussion, is the most common type of traumatic brain injury [1, 2]. The Mild Traumatic Brain Injury Committee of the American Congress of Rehabilitation Medicine [3] describes mTBI as a mild insult to the head that results in a brief period of unconsciousness followed by impaired cognitive function. Along with impaired cognitive function, mTBI causes an array of symptoms, most notably headaches, fatigue, depression, anxiety and irritability, collectively referred to as post-concussion syndrome (PCS)[3]. The time it takes for symptoms to resolve in the majority of individuals is approximately 3 months, however, some individuals continue to experience symptoms beyond 1 year post-injury [4, 5]. Those with persistent symptoms are said to experience persistent PCS [5, 6]. While persistent PCS has been defined numerous ways in the literature, generally it includes the presence of the aforementioned symptoms, including cognitive impairment. As initially reported by Rutherford et. al., persistent PCS is estimated to impact 15% of individuals with a first-time concussion [7–9].

Amongst the many sequelae of mTBI, cognitive impairment may be paramount in relation to its contribution to long-term dysfunction [10]. Impairment in numerous cognitive domains has been reported in mTBI, including executive function, learning and memory, attention and processing speed, among others [10]. Evidence indicates that a single concussion can disrupt the neurological mechanisms underlying cognition [11]. The impairment is robust and therefore readily detectable in the early phase post-injury, but the long-term outcomes are unclear largely due to a dearth of research. It is well established that a single mTBI results in pathophysiological changes in the brain. Included amongst these pathophysiological changes is altered white matter structure and function (e.g., diffuse axonal injury, DAI) as well as the so called ‘neurometabolic cascade’ that is characterized by altered neurotransmitter activity and subsequently altered levels of brain excitability [12–14]. While not observed using conventional imaging, DAI has been found in numerous brain regions following a single mTBI using diffusion tensor imaging (DTI) [15–18]. Abnormal integrity of white matter tracts has even been observed in the absence of a clinical diagnosis of concussion [19]. Given that even a single mTBI induces pathophysiological changes in the brain that can be detected in both the acute and chronic phases post-injury, one might anticipate these pathophysiological changes manifesting as cognitive impairment. As such, why the incidence of cognitive impairment is not higher than that reported for PCS (i.e., 15%) is not apparent. As PCS is defined as a collection of symptoms (e.g., requiring 3 of 8 symptom categories as reported in Daneshvar and collegues [5]), it is difficult to identify the long-term incidence of specific symptoms resulting from mTBI, including cognitive impairment.

To date, the studies that assess long-term cognitive outcomes in singly-concussed individuals have not been gathered and reviewed. To address this gap in knowledge, we performed a scoping review of the literature reporting cognitive outcomes in first-time concussed individuals in the chronic phase (i.e., > 3 months post-injury) to determine the impact of a single mTBI. Establishing that even a single concussion has long-term impact on cognitive function will add support to the notion that ‘mild’ traumatic brain injury is anything but.

## Methods

### Type of review

Given the study purpose, we performed a scoping, as opposed to systematic, review. As defined in Colquhoun and colleagues [20], a scoping review is “a form of knowledge synthesis that addresses an exploratory research question aimed at mapping key concepts…in research related to a defined area or field by systematically searching, selecting and synthesizing existing knowledge” (p. 1292–94), whereas a systematic review is intended to determine what is known in a given area of research with a focus on making recommendations for clinical practice [21]. As detailed below, we followed the framework put forth by Arksey and O’Malley (and later revised by Levac and colleagues) to perform the review [22, 23]. Consistent with this framework, our study included a descriptive numerical summary and qualitative approach, as opposed to a quantitative statistical one [20].

### Scoping search

A broad search of the literature was performed to identify all keywords and search terms for two concepts: concussion and cognitive impairment. Three electronic databases, CINAHL, Embase, and Medline/Ovid were used for this scoping review. Prior to conducting the search, the keywords and search terms were organized into a search translation table (see Table 1). The search translation table organizes both keywords and controlled vocabulary terms to assist in maintaining equivalent searches across the three databases. Each controlled vocabulary term for all three databases was exploded to include related terms. For the purposes of this review, we operationally defined cognitive impairment as any impairment to the cognitive processes related to executive function. Controlled vocabulary terms included the cognitive domains of “learning” and “memory”. Given the inextricable relationship between learning and memory and the various cognitive domains (i.e., executive function, attention, processing speed, and language function), we did not believe the controlled vocabulary would pose any limitations as the search criteria permitted inclusion of all types of cognitive testing, regardless of their respective cognitive domains.

**Table 1:** Search translation table.

CINAHL	EMBASE	Medline
Controlled Vocabulary Terms*		
**Concept 1: Concussion**		
(MH "Brain Concussion")	'brain concussion'/exp	exp brain concussion/
(MH "Postconcussion Syndrome")	'postconcussion syndrome'/exp	exp post-concussion syndrome/
**Keywords & Phrases**		
(mild N5 (head OR crani* OR cerebr* OR brain* OR skull* OR hemispher* OR intra?cran* OR inter?cran* OR intracran* OR intercran* OR "diffuse axonal") N3 (injur* OR trauma* OR damag* OR? edema* OR contusion* OR concus*))	mild NEAR/5 (head OR crani* OR cerebr* OR brain* OR skull* OR hemispher* OR intra?cran* OR inter?cran* OR intracran* OR intercran* OR 'diffuse axonal') NEAR/3 (injur* OR trauma* OR damag* OR? edema* OR contusion* OR concus*)	(mild adj5 (head or crani* or cerebr* or brain* or skull* or hemispher* or intra?cran* or inter?cran* or intracran* or intercran*) adj3 (injur* or trauma* or damag* or oedema* or edema* or contusion* or concus*)).ab,ti.
Controlled Vocabulary Terms*		
**Concept 2: Cognitive Impairment**		
(MH "Neurobehavioral Manifestations+")	mild cognitive impairment'/exp	exp mild cognitive impairment/
(MH "Memory+")	memory'/exp	exp memory/
(MH "Learning+")	learning'/exp	exp learning/
**Keywords & Phrases**		
(Learn* OR memor* OR neurobehavio* OR cogniti* OR neurologi*) N3 (Impair* OR deficit* OR disturb* OR impact* OR disorder* OR outcome*)	(learn* OR memor* OR neurobehavio* OR cogniti* OR neurologi*) NEAR/3 (impair* OR deficit* OR disturb* OR impact* OR disorder* OR outcome*)	((learn* or memor* or neurobehavio* or cogniti* or neurologi*) adj3 (impair* or deficit* or disturb* or impact* or disorder* or outcome*)).mp.

*Controlled Vocabulary Terms: CINAHL = CINAHL Headings, EMBASE = Emtree terms, and Medline/Ovid = Medical Subject Headings (MeSH) terms

The scoping search was performed on July 25^th^, 2015. The search yielded 5900 citations, 579 from CINAHL, 2167 from EMBASE, and 3154 from Medline/Ovid. The 5900 citations were exported into a reference manager database (Mendeley). After the duplicates were removed, 3741 citations remained.

### Refining the literature—Phases 1 & 2

**Fig 1:**
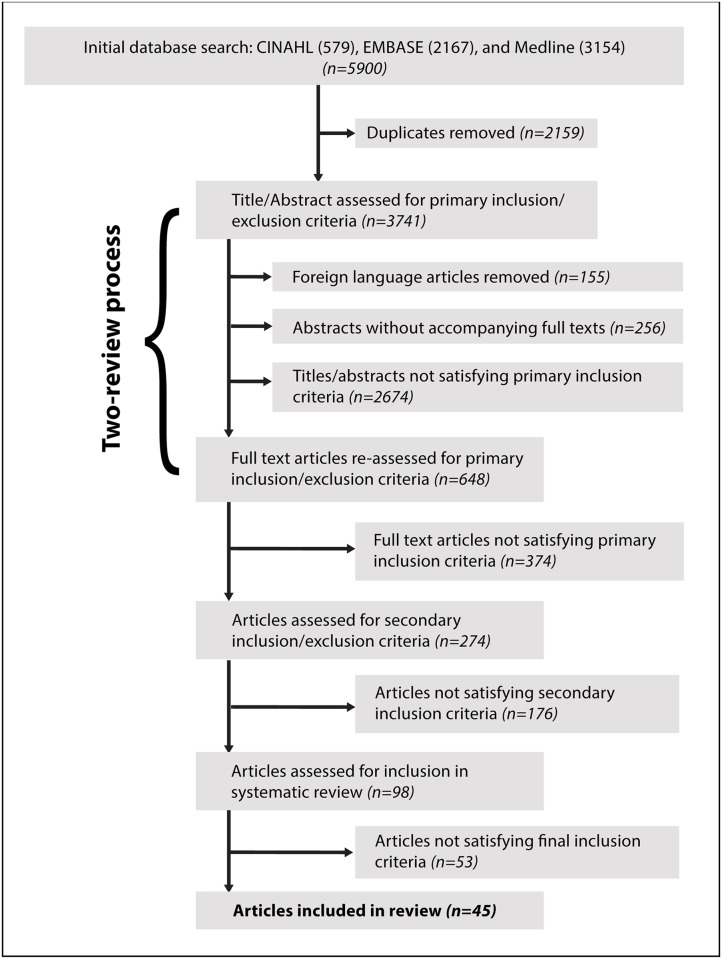
*F*low diagram representing each stage of the article selection process of the scoping search and citation review.

Fig 1 illustrates the search process and application of the study inclusion/exclusion criteria. The process for selecting which studies to include was broken down into four phases. In the first phase, two independent reviewers assessed the title and abstract of each of the 3741 citations, indicating their decision for inclusion/exclusion in an Excel spreadsheet (Microsoft Office, 2015) based on the primary inclusion/exclusion criteria outlined in Table 2. Briefly, included citations had to have human participants with chronic (i.e., ≥3 month post-injury interval) mTBI that underwent any form of cognitive testing. A third reviewer resolved any disagreements amongst the two reviewers regarding study inclusion/exclusion. As illustrated in Fig 1, 648 citations remained following phase 1.

**Table 2:** Inclusion/Exclusion criteria for each selection phase process.

Phase	Inclusion Criteria	Exclusion Criteria
1: Titles/abstracts reviewed*	Human participants with chronic (post-injury interval of ≥3 mo.) mild TBI Participants tested for cognitive impairments using neurocognitive testing	Foreign language articles Articles without accompanying full texts (i.e., conference abstracts/posters) Subjective questionnaires used for cognitive testing
2: Full-text articles reviewed	Same as above	Same as above
3. Full-text articles reviewed	Participants assessed at discrete time points post-injury (i.e., exclude studies only reporting on mean/SD for post-injury interval) Specific number of concussions reported (within 1 concussion)	Participants suspected of malingering cognitive deficits or those involved in litigation for their injuries
4. Post-analysis	Participants with a history of a single concussion	Studies recruiting participants based on their positive mTBI symptomology Participants with multiple or lifetime incidence of concussions

*Two-reviewer process

Phase 2 replicated phase 1, with a single reviewer re-assessing the full texts to ensure adherence to our primary inclusion/exclusion criteria (Table 2). As illustrated in Fig 1, 274 full-text articles remained following phase 2 review. Throughout phase 2, data were extracted from each article that satisfied the primary inclusion/exclusion criteria, including: number and age of participants, mTBI mechanism of injury (e.g., blast related versus motor-vehicle accident [MVA]-induced), concussion history (e.g., number of previous concussions, time since last concussion), cognitive test(s)/subtest(s) used to assess cognitive impairment, participant’s litigation status and/or suspected malingerers, and use of treatment/intervention (e.g., hyperbaric oxygen treatment). Other pertinent information such as comorbidities (e.g., PTSD, depression, Alzheimer’s disease) was also noted. Data from treatment/intervention studies were limited to the pre-treatment or pre-intervention time points. In other words, we only used baseline scores on cognitive assessments for participants being tested on their cognition following a treatment/intervention to ensure no confounding effects of the treatment /intervention on our results.

### Refining the literature—Phase 3

In the third phase of review, we re-assessed the remaining articles with a second set of inclusion/exclusion criteria (Table 2). To assess the long-term cognitive outcomes of mTBI with temporal specificity (i.e., precise post-injury intervals), we only included articles that performed assessments of cognitive function at discrete time points post-injury. Thus, we excluded studies that only report a mean and/or range of post-injury intervals for a group of individuals with mTBI. We opted to include studies reporting only means or ranges of post-injury intervals if the mean and/or range corresponded to a post-injury interval of greater than 5 years. The reasons for this exception are twofold. First, cognitive outcomes will not continue to improve long after the most recent injury—cognitive outcomes after the first five years will likely not change in the next five (or more) years [24, 25]. In other words, the precision of the post-injury interval becomes less relevant in the longer term. Second, the majority of studies reporting long-term cognitive outcomes in individuals with mTBI are not often temporally specific with respect to post-injury intervals. Excluding these studies would greatly diminish our ability to comprehensively review the literature reporting on long-term cognitive outcomes in mTBI.

In order to assess the relationship between the number of previously sustained concussions and cognitive function, we also chose to exclude studies that only specify a range and/or mean number of concussions. Thus, studies reporting that their participants sustained, for example, between 1–5 concussions would be excluded from our analysis. Studies noting a range of concussions within 1 (i.e., between 1–2 concussions) were included. This exception, like that for the post-injury interval, minimizes the number of studies excluded, ensuring that our review is comprehensive so that we can better synthesize the wide breadth of research.

During phase 3, we also excluded participants who were engaged in litigation associated with their injury, or those suspected of malingering (i.e., exaggerating or fabricating) their cognitive deficits. The exclusion of these participants ensures our sample is not confounded with individuals who have an incentive to perform poorly on the cognitive outcome measures. Following phase three review, 98 articles remained.

### Further refining the literature—Phase 4

The fourth phase of article selection included a set of inclusion/exclusion criteria (see Table 2) that we developed following an examination of the data extracted in the prior phases. Specifically, we assessed the homogeneity of the 98 articles remaining after phase 3 with respect to the following variables: number of concussions sustained; outcome measures used to assess cognitive impairment; method of participant recruitment (i.e., whether the participants were recruited based on their positive symptomology of cognitive impairment); and method for determining cognitive impairment (i.e., comparison groups, author-defined normative data, or author-provided cut-off scores on given outcome measures). In conducting this analysis, we found that the majority of the participants (i.e., 4196 of 4239) had a history of a single concussion while only 43 participants had a history of more than one concussion (i.e., 2 with 2 mTBIs, 1 with 3 mTBIs, 39 with 4 mTBIS, and 1 with 5 mTBIs). Given the disproportional spread of the data with respect to concussion history (a direct result of the search strategy design), we focused our analysis on the cognitive outcomes in individuals with a history of a single concussion. Thus, in our final exclusion criteria outlined in the last row of Table 2, we excluded studies examining cognitive outcome measures in individuals with a history of multiple concussions or lifetime concussion exposure. In order to minimize exclusion, we chose to include studies where the participants were likely (but not certainly) first-time concussed. Those included studies that: (1) did not specify whether their participants were exclusively singly concussed or (2) did not exclude participants based on their history of a previous concussion. Nevertheless, we included this as a variable in our data analysis, as elaborated on in the results section.

During our preliminary examination of the data, we also found that several studies had specifically recruited their participants on the basis of their persisting cognitive symptoms. This creates an unacceptable bias, as these studies would artificially exaggerate the presence of persisting cognitive impairment among the average singly concussed participant. Thus, we excluded case studies and other studies recruiting participants for positive symptomology. Finally, our preliminary examination of the data also revealed that not all of the studies presented their data in a way that would facilitate the dichotomization of participants into cognitively impaired and cognitively unimpaired groups (see below for Methods on dichotomization process). Thus, we decided to only include studies that included comparison groups (i.e., healthy controls or trauma controls), normative data, or cut-off scores on cognitive outcome measures. Following these exclusions, there were 45 studies remaining for the final scoping review (Fig 1, Tables 3–6).

**Table 3:** Study information for all participants at 3 months post-injury.

CI	Study	N	Control/Method of Comparison	Age (M, SD)	mTBI Definition	C/UnC	# mTBIs
CI	Rieger et al., [26]	39	OI: A/S/R	8–17 yr.	Standard (GCS = 14–15)	UnC	1^a^
	Phillipou et al., [27]	26	HC: A	12.8 (2.1)	Standard	—	1^b^
	Tay et al., [28]	31	A/S/E/R	40.6 (14.7)	Standard (LOC < 20 min)	UnC	1^c^
	Kwok et al., [29]	15	HC: A/S/E	38.6 (12.4)	Standard	C	—
	Su et al., [30]	54	Cut-off scores	39.8 (0.7)	Standard	—	1^a^
	Siman et al., [31]	17	HC: A/S/E	20.2 (5.4)	Standard	—	1^b^
	Ponsford et al., [32]	90	Trauma controls	35.0 (13.1)	Standard	UnC	—
	Paré et al., [33]	37	A/S/E	26.7 (10.3)	Standard	—	1^d^
	Kinsella et al., [34]	50	OI & HC: A/S/E	76.5 (7.6)	Standard	C	1^b^
	Marsh & Smith [35]	15	A/E	27.1 (12.6)	"Diagnosis of concussion"; LOC < 20 min	UnC	1^f^
	Xu et al., [36]	40	Cut-off scores	39.3 (13.1)	Standard	UnC	1^a^
	De Boussard et al., [37]	29	Normative data	37.2 (*NA*)	Standard (GCS = 14–15)	C	—
	Hanten et al., [38]	59	OI & HC: A/S/R/SES	18.2 (4.6)	Standard	UnC	1^b^
	Heitger et al., [39]	37	A/S/E	29.1 (12.7)	Standard	UnC	1^e^, ^**α**^
	Bohnen et al., [40]	8	Normative data	27.2 (14.0)	Standard (GCS = 15)	UnC	1^a^
	Rotarescu & Ciurea [41]	96	Normative data	10.5 (3.4)	GCS = 14–15 w amnesia	—	—
CU	Ponsford et al., [42]	119	HC: A/S/E/SES	11.3 (2.9)	Standard	—	≥1^c^
	Su et al., [30]	159	Cut-off scores	39.8 (0.7)	Standard	—	1^a^
	Ponsford et al., [43]	84	HC: A/S/E/SES	26.4 (13.9)	Standard	—	≥1^c **α**^
	Xu et al., [36]	78	Cut-off scores	39.3 (13.1)	Standard	UnC	1^a^
	De Boussard et al., [37]	68	Normative data	37.2 (NA)	Standard (GCS = 14–15)	C	—
	Maillard-Wermelinger et al., [44]	186	OI: A/S/E/SES	12.0 (2.2)	Standard	C	1^b^
	Bohnen et al., [40]	33	Normative data	27.2 (14.0)	Standard (GCS = 15)	UnC	1^a^
	Levin et al., [45]	36	A/S	9.8 (3.1)	GCS = 13–15	—	—

**A**: Age; **C**: Complicated **E**: Education; **GCS**: Glasgow Coma Scale; **HC**: Healthy Controls; **LOC**: Loss of Consciousness; **OI**: Orthopedic Injury Control; **S**: Sex; **SES**: Socioeconomic Status; **UnC**: Uncomplicated

1^a^: No previous TBI

1^b^: No previous TBI requiring hospitalization

1^c^: Previous head injuries not excluded

1^d^: No previous TBI resulting in the loss of consciousness for >5 min

1^e^: No previous TBI with persisting symptoms

1^f^: No previous TBI requiring hospitalization in the last 6 mo.

**Table 4:** Study information for all participants at 6 months post-injury.

CI	Study	N	Control/Method of Comparison	Age (M, SD)	mTBI Definition	C/UnC	# mTBIs
CI	Phillipou et al., [27]	26	HC: A	12.8 (2.1)	Standard	—	1^b^
	Wong et al., [46]	4	A/S/E	52 (17.9)	Standard	UnC	1^a^
	Muller et al., [47]	19	Defined norms	35.1 (—)	GCS 13–15; LOC/retrograde amnesia	C	—
	Ellemberg et al., [48]	10	A/S/E/Sport*	22.7 (—)	AAN Grade II concussion	—	—
	Miles et al., [16]	4	Cut-off scores	33.4 (—)	Standard	UnC	1^a^
	Wrightson et al., [49]	59	A/S/SES	3.38	“Mild head injury” diagnosis	—	1^a^
	Heitger et al., [39]	37	A/S/E	29.1 (12.7)	Standard	UnC	1 ^a, **c**^
	Bohnen et al., [40]	7	Normative Data	27.2 (14.0)	Standard (GCS = 15)	UnC	1^a^
	Babikian et al., [12, 50]	36	Normative Data	12.7 (2.0)	Standard; AIS level 1–2	—	≥1
	Rotarescu & Ciurea [41]	96	Normative data	10.5 (3.4)	GCS = 14–15 with amnesia	—	—
CU	Muller et al., [47]	36	Normative Data	35.1 (—)	GCS 13–15; LOC/retrograde amnesia	C	—
	Miles et al., [16]	8	Cut-off Scores	33.4 (—)	Standard	UnC	1^a^
	Barrow et al., [51]	28	A/E/R	41 (—)	Standard	UnC	1^a^
	Bohnen et al., [40]	34	Normative Data	27.2 (14.0)	Standard (GCS = 15)	UnC	1^a^
	Babikian et al., [12, 50]	88	Normative Data	12.7 (2.0)	Standard; AIS level 1–2	—	≥1

**A**: Age; **AAN**: American Academy of Neurology; **AIS**: Abbreviated Injury Score; **C**: Complicated **E**: Education; **GCS**: Glasgow Coma Scale; **HC**: Healthy Controls; **LOC**: Loss of Consciousness; **S**: Sex; **SES**: Socioeconomic Status; **UnC**: Uncomplicated. AAN Grade II concussion: No LOC, transient confusion, concussion symptoms, or mental status abnormality lasting more than 15 minutes.

1^a^: No previous TBI

1^b^: No previous TBI requiring hospitalization

1^c^: No previous TBI with persisting symptoms

* Sport matched for type and length of involvement

**Table 5:** Study information for all participants at 12 months post-injury.

CI	Study	N	Control/Method of Comparison	Age (M, SD)	mTBI Definition	C/UnC	# mTBIs
CI	Catale et al., [52]	15	A/S/E/SES	8.3 (1.3)	GCS = 15; LOC < 10 min; PTA < 1 hr.	UnC	1^a^
	Lee et al., [53]	28	A/S/E	30.2 (8.0)	Standard	C	1^a^
	Polissar et al., [54]	53	A/S/E/SES	*"Children"*	GCS = 13–15	C	—
	Kashluba et al., [55]	102	Normative data	48.6 (16.4)	Standard	C	—
	Romero et al., [56]	49	Normative data	30.9 (12.4)	Standard	C	1^a^
	Stålnacke et al., [57]	69	A/S/E	40.9 (19.5)	GCS = 13–15; LOC < 30 min.	UnC	1^c^
	Chadwick et al., [58]	29	A/S/SES	9.6 (2.5)	1 hour < PTA < 7 days	C	—
	Wrightson et al., [49]	57	A/S/SES	3.38	“Mild head injury” diagnosis	—	1^a^
	Heitger et al., [39]	37	A/S/E	29.1 (12.7)	Standard	UnC	1^d^
	Anderson et al., [59]	17	A/S/SES	5.1 (1.5)	GCS = 13 = 15; “alteration of consciousness”	UnC	1^a^
	Babikian et al., [12, 50]	21	Normative Data; OI: A/S/E/SES	12.7 (2.0)	Standard; AIS level 1–2	—	≥1
	Rotarescu & Ciurea [41]	96	Normative data	10.5 (3.4)	GCS: 14–15 w amnesia	—	—
CU	Wäljas et al., [60]	103	A/S	37.8 (13.5)	Standard	C	—
	Dikmen et al., [61]	157	TC: A/S/E	28.1 (11.1)	GCS = 13–15	C	—
	Zhou et al., [62]	19	A/S/E	34 (11.5)	Standard	UnC	1^a^
	Croall et al., [63]	18	A/S/E	33.9 (14.8)	Standard	—	—
	Maillard-Wermelinger et al., [44]	186	OI: A/S/E/SES	12.0 (2.2)	Standard	C	1^b^
	Babikian et al., [12, 50]	55	Normative Data	12.7 (2.0)	Standard; AIS level 1–2	—	≥1
	Jaffe et al., [64]	40	A/S/E/SES	6–15 yrs	“Mild head injury with LOC”	—	1^b^
	Levin et al., [45]	36	A/S	9.8 (3.1)	GCS = 13–15	—	—

**A**: Age; **AIS**: Abbreviated Injury Score; **C**: Complicated **E**: Education; **GCS**: Glasgow Coma Scale; **LOC**: Loss of Consciousness; **OI**: Orthopedic Injury Control; **PTA**: Post-Traumatic Amnesia; **S**: Sex; **SES**: Socioeconomic Status; **UnC**: Uncomplicated

1^a^: No previous TBI

1^b^: No previous TBI requiring hospitalization

1^c^: Previous head injuries not excluded

1^d^: No previous TBI with persisting symptoms

**Table 6:** Study information for all participants at >12 months post-injury.

	Study	PII (Yr.)	N	Control/Method of Comparison	Age (M, SD)	mTBI Definition	C/UnC	# mTBIs
CI	Mangels et al., [65]	1.5	10	A/S/E	29.4 (3.3)	GCS = 13–15	C	—
	Chadwick et al., [58]	2.25	29	A/S/SES	9.6 (2.5)	1 hour < PTA < 7 days	C	—
	Anderson et al., [59]	2.5	17	A/S/SES	5.1 (1.5)	GCS = 13 = 15; “alteration of consciousness”	UnC	1^a^
	Mangels et al., [65]	3.7	11	A/S/E	29.4 (3.3)	GCS = 13–15	C	—
	Wrightson et al., [49]	3–4	57	A/S/SES	3.38	“Mild head injury” diagnosis	—	1^a^
	McCauley & Levin [66]	5	17	OI: A/S/SES	15.3 (2.1)	GCS = 13–15	C	—
	Geary et al., [67]	5	40	A/S/E	29.6 (1.7)	Standard	UnC	—
	Konrad et al., [68]	6	14	A/S/E	36.7 (12.4)*	Standard	C	1^a^
	Vanderploeg et al., [69]	8	254	MVA & HC: A/E/R	37.8 (2.5)	“mTBI with LOC”	—	—
CU	Jaffe et al., [64]	3	40	A/S/E/SES	6–15 yr.	“Mild head injury with LOC”	—	1^b^
	Konrad et al., [68]	6	19	A/S/E	36.7 (12.4)*	Standard	C	1^a^

**A**: Age; **C**: Complicated **E**: Education; **GCS**: Glasgow Coma Scale; **HC**: Healthy Controls; **LOC**: Loss of Consciousness; **MVA**: Motor-vehicle accident; **OI**: Orthopedic Injury Control; **PTA**: Post-Traumatic Amnesia; **S**: Sex; **SES**: Socioeconomic Status; **UnC**: Uncomplicated

1^a^: No previous TBI

1^b^: No previous TBI requiring hospitalization

* Time of testing

### Addressing the research objective

To address our research objective of investigating the impact mTBI has on cognitive function long after a single concussive injury, we examined the information pertaining to concussion history (i.e., post-injury interval and number of previous concussions) and cognitive outcomes (i.e., presence versus absence of cognitive impairment). In order to make inferences about cognitive function, we dichotomized participants, assigning them the status of either “cognitively unimpaired” (CU) or “cognitively impaired” (CI), for each cognitive outcome measure and post-injury interval at which an assessment of cognitive function was performed. Cognitive impairment status was assigned to groups of participants based on group outcome measure data. An assignment of CU/CI was made using one of three comparison scores, including: 1) studies that provided outcome measure data from control groups (i.e., healthy controls or trauma controls); 2) studies that provided normative data for a given outcome measure; or 3) studies that provided cut-off scores for a given outcome measure. Thus, groups of participants were classified as CI if their outcome measure score significantly differed from those of the control groups or the normative data, or if they were below author-identified cut-off scores.

A final consideration must be addressed regarding the process of dichotomizing participants into CU/CI groups. Since the majority of the included studies assessed groups of participants using multiple outcome measures, we defined “CI” as participants that show impairment on *any* outcome measure. In other words, if a participant shows impairment on 1 of 3 outcome measures, they were assigned to the CI group. Since our study is primarily concerned with demonstrating *any* form of cognitive impairment, it is not important if their impairment only manifests on one outcome measure; an individual who is impaired on one function still exhibits cognitive impairment.

## Results

### Global cognitive impairment

Information pertaining to each CI/CU group was extracted from each study and summarized in Tables 3–6. Specifically, Tables 3–6 present the following information: (1) the number of participants cognitively impaired or unimpaired at each post-injury interval; (2) the method used to determine cognitive impairment (i.e., comparison groups, author-provided normative data, or author-provided cut-off scores for a given outcome measure); (3) the mean age and SD of the participants; (4) how the authors defined mTBI (note: “Standard” refers to three criteria: Glasgow Coma Scale (CS) = 13–15, a Loss of Consciousness (LOC) < 30 minutes, and a post-traumatic amnesia (PTA) < 24 hours); (5) whether the participants had complicated (i.e., presence of radiological findings not including a linear skull fracture) or uncomplicated mTBI; and (6) the participant inclusion criteria given for number of previous concussion.

From Tables 3–6, it is apparent that the studies included in our scoping review were not homogeneous with respect to any of the outlined variables. For example, while we included studies that used three different methods of comparison for determining cognitive impairment (i.e., comparison groups, normative data, and cut-off scores), there was variability within the comparison groups. Some studies used a healthy control group while others used either an orthopedic injury control group or a trauma control group. Further, those that did use a healthy control group may have included different variables that were equivalent across groups (i.e., any combination of the following: age-matched, sex-matched, education-matched, and socioeconomic status-matched controls). Similarly, the studies did not all adhere to one definition of mTBI. The majority of studies used the standard definition (i.e., GCS 13–15, LOC < 30 min, PTA < 24 hours)[3], however, some studies either adhered to a variation of the standard definition (i.e., standard definition with the exception of a GCS = 14–15) or an entirely different definition (i.e., PTA > 1 hour and < 24 hours). Further to the information presented above, it is also apparent from Tables 3–6 that the studies included in our review were not consistent in their inclusion or exclusion of participants with complicated mTBI (i.e., mTBI with presence of neuroradiological findings). Some studies included those with complicated mTBI, others excluded them, and the remaining studies failed to provide this information. Finally, Tables 3–6 also show that the studies in our review were not consistent regarding their inclusion/exclusion criteria of participants with previous mTBIs. Interestingly, 18 studies did not specify if their participants had sustained a previous mTBI. It is thus possible that the participants in these studies were not first-time concussed. For this reason, and since some studies did not specifically exclude those with previous concussions, we included this variable in our data synthesis (discussed below).

**Fig 2:**
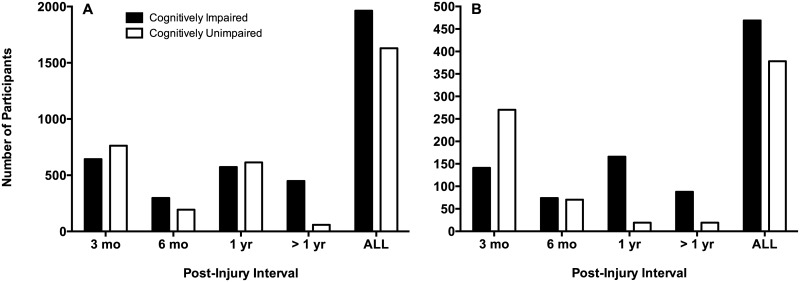
Incidence of cognitively impaired (black bars) and unimpaired (white bars) individuals at various time points post-injury from studies reporting cognitive outcomes using either author-supplied normative data or comparison groups (i.e., healthy or trauma controls) for the entire sample (A) and in individuals with a confirmed history of a single concussion only (B).

Fig 2A illustrates the overall incidence of cognitive impairment in individuals with mTBI at various post-injury intervals for all studies included in our scoping review. Fig 2B illustrates the overall incidence of cognitive impairment at the same post-injury intervals, however, for participants who had a reported history of a single concussion only. In other words, Fig 2B includes only those studies that excluded participants with previous mTBI. This criteria is represented in the final column of Tables 3–6 as 1^a^, or “no previous TBI”. The results from each post-injury interval are collapsed together in the final cluster of columns in each Fig 2A and 2B to yield a total number of participants who show long-term cogntive impairment across all studies and all time points in this review. It is important to note, however, that participants who were tested across multiple time points could be accounted for more than once in Fig 2. For example, prospective studies that assess participants at say, both 3- and 6-months post injury would be represented at both time points in Fig 2. Thus, when we collapse all post-injury intervals in the last cluster of columns, participants from those studies will have been accounted for more than once.

Fig 2 demonstrates that the incidence of individuals who show persitent cognitive impairment following an mTBI is much higher than previous estimates (i.e., around 15%) reported in the literature for PCS [4, 6–9, 70]. Specifically, 1963 participants out of 3593, or approximately 55% of our sample collapsed across all time points showed cognitive impairment. After filtering out the studies that did not ensure their participants were first-time concussed (Fig 2B), we still show 55% of our participant sample collapsed across all time points were cognitively impaired (i.e., 469 participants out of 847). Thus, Fig 2B demonstrates that the high incidence of long-term cognitive impairment in our results cannot be attributed to the possibility that a subset of participants in Fig 2A may have experienced more than one mTBI. Our results do not hint at a temporal relationship of cognitive impairment wherein participants were less likely to be cognitively impaired at later post-injury intervals. This is evident in both Fig 2A and 2B in that the incidence of cognitive impairment was not associated with time post-injury—however, our participant sample was not restricted to prospective and longitudinal study designs. Specifically, Fig 2A demonstrates that 46% of the participant sample was cognitively impaired at 3 months, 61% at 6 months, 48% at 12 months, and 88% at >12 months post-injury. We do not take the particularly high percentage of participants that were cognitively impaired at the >12 months post-injury interval to show that individuals are more likely to be cognitively impaired after 12 months. Instead, this finding is likely attributable to the limited number of studies assessing individuals past one year.

**Fig 3:**
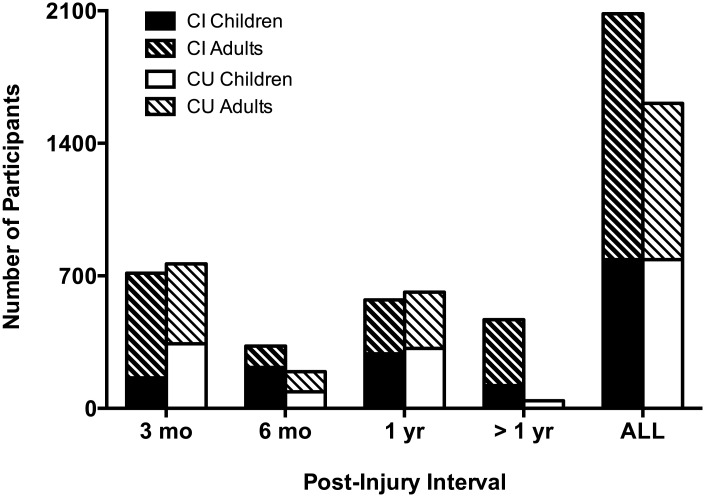
Incidence of cognitively impaired (black bars) and unimpaired (white bars) individuals separated into children (no pattern) and adults (pattern).

To determine whether our results were similar in both children (<18 years) and adults (≥18 years), we present the data from Fig 2A again in Fig 3, this time including age as a third variable. On visual inspection, it does not appear that age had any impact on the high incidence of long-term cognitive impairment in individuals with mTBI. While there does appear to be many more adults in the CI group than in the CU group at the >12 months post-injury interval, this is likely due to the limited number of studies we had reporting cognitive outcomes at this time interval. The last cluster of columns in Fig 3 can be quantified as follows: 786 children with cognitive impairment; 786 children without cognitive impairment; 1177 adults with cognitive impairment; and 844 adults without cognitive impairment. In other words, 50% of the children and aproximately 58% of the adults in our scoping review showed some form of cognitive impairment.

## Discussion

The last several decades of mTBI research have seen an expansion in our understanding of the long-term cognitive and behavioural consequences. Whereas mTBI used to be thought of as a relatively inconsequential “mild” injury, it is now more closely associated with the latter three letters of its acronym—“traumatic brain injury”. This shift in our understanding is owing to several revelations in mTBI research. Namely, researchers have shown that both single and multiple mTBI(s) induce pathophysiological changes in the brain that can be detected in both the acute and chronic phases post-injury. They have also shown how these pathophysiological changes manifest as measurable cognitive impairment in both single or multiple mTBI(s) [11, 71]. While studies assessing singly-concussed individuals consistently show impairment early (3 months) post-injury, it has been suggested that only 15% of those individuals will go on to experience persistent symptoms in the chronic phase post-injury (i.e., persistent PCS)[7–9]. Given our understanding of the underlying pathophysiological consequences of mTBI in the chronic phase (i.e., DAI and the neurometabolic cascade), it is surprising that the literature has not reported a greater portion of individuals with cognitive impairments in the chronic phase (i.e., considerably more than 15%). For this reason, our scoping review assessed the evidence in the mTBI literature for cognitive impairment in singly-concussed individuals long after the injury (i.e., in the chronic phase post-injury).

The main finding from our scoping review relates to the incidence of persistent cognitive impairment in individuals with chronic stage mTBI following a single concussion. The findings from our scoping review do not support the conclusions of previous reports that a single mTBI leads to PCS in 15% of individuals in the chronic stage injury, and that the other 85% will see resolution of symptoms during the acute phase [7–9]. In contrast, we show that a large proportion of individuals with a single mTBI will continue to demonstrate measurable impairment in various cognitive domains including executive function, learning/memory, attention, processing speed, and language function long after the initial injury. Further, we show that our finding holds true in our sample of both children and adults (Fig 3), and in studies both controlling for, and failing to control for, previous concussion exposure (Fig 2). While the methods used in this scoping review are not appropriate for determining the precise incidence of persistent cognitive impairment following mTBI, our results highlight a major contradiction in the mTBI literature. While the 15% estimate for PCS is widely reported in the mTBI literature, our results suggest that for cognitive impairment, this value may well be a gross underestimation of the true incidence. But how does the current sample of participants compare to prior work from which the 15% estimate arose? Rutherford (1977) described the initial sample of participants (all ≥ 12 yrs of age) as being first time concussed, where concussion was defined as “a period of amnesia resulting from a blow to the head” [72]. Initial estimates indicated at 6 weeks post-injury, 49% were symptom free, 39% reported between 1 and 6 symptoms, and 2% report 6 or more. In a sub-sample of these participants (as reported in Rutherford et. al., 1979) examined at one-year post injury, 15% reported the presence of symptoms [7]. While the contemporary definition of concussion is far more nuanced than that reported in these prior studies, review of Tables 3–6 woud indicate all of the studies included in this review (at a minimum) conform to a standard definition of mTBI which includes a loss of consciousness and PTA < 24 hrs following the injury.

While numerous reports cite the incidence of PCS as being 15% [7–9], the primary research demonstrating this finding suffers from several limitations. First, those studies have relied on methods that may be insufficiently sensitive to detect subtle changes to cognition following mTBI. For instance, studies examining singly-concussed individuals in the chronic phase post-injury have been able to detect cognitive impairment on neurophysiological correlates of cognitive function such as brain activity (i.e., event-related potentials obtained via electroencephalography) whereas standard assessments of cognitive function did not show any impairment [73]. In other words, cognitive impairments may persist undiagnosed owing to our limited ability to detect them using standard behavioural assessments [73, 74]. Similarly, this prior work often focused on symptoms (e.g., anxiety, loss of concentration) that could be linked to cognitive impairment as opposed to cognitive impairment itself. Despite this discord in assessment, the 15% estimate appears to be generalized to PCS and other mTBI-related impairments in the literature. Alternatively, and as discussed earlier, it may be that clustering cognitive impairment as one of several symptoms required for a diagnosis of PCS greatly reduces the incidence in which cognitive impairment is reported in the literature. Regarding the sensitivity of outcome measures, as our study relied on reviewing the evidence from research that has used these very methods, it was not designed to overcome this limitation. By unpacking cognitive impairment as a single symptom however, this work did overcome other limitations that may have contributed to the 15% estimate being an underestimation of the cognitive costs associated with mTBI. Additionally, performing a scoping review overcomes single-study limitations such as low power, limited numbers of participants, and lack of generalizability of the study’s sample population. Moreover, our study was able to assess cognitive outcomes at multiple time points when the majority of the individual studies only examined one post-injury interval. Given the inability of our study to overcome the limitation of insufficiently sensitive methodology used to assess cognition, it is possible that our results represent a further underestimation of the incidence of persistent cognitive impairment following a single mTBI.

### Limitations

There are several limitations to the current work that should be considered when interpreting the results. The first major limitation pertains to the article selection process used. Our exclusion of studies reporting only group data for post-injury interval or number of concussions greatly decreased the sample size. Including these studies, however, would have greatly increased heterogeneity and thus increased the difficulty of pooling data across studies. Further, we would not have been able to temporally organize our data (i.e., with respect to post-injury interval) had we included studies reporting mean post-injury intervals. Unfortunately, the mTBI literature has not emphasized the reporting of individual participant data for post-injury intervals or number of previous concussions. This artefact of the mTBI literature suggests that the primary interest of mTBI research has not been on establishing the relationship between post-injury interval and the amelioration of cognitive symptoms. This relates to another limitation of our work—the participants in our review were not all gathered from longitudinal studies assessing the same participants across each post-injury interval. Solely looking at data from longitudinal studies, however, would have greatly diminshed our sample size.

We included studies using three different methods of comparison for assessing outcome measures—that is, those using normative data, those using cut-off scores, and those providing control groups. While the control group method of comparison is applied to group data, the cut-off score and normative data methods were applied to individual data. Thus, for studies providing control groups, the entire mTBI group would be assigned to either the CI/CU group whereas studies providing cut-off scores or normative data, individual participants were allocated to each CI/CU group. Individual participant binarization is not prone to the limitations posed by group data binzarization using control groups. Group data binarization inevitably bins groups of participants together disregarding the individual data on outcome measures. Despite the obvious limitation of working with group data, excluding these studies would have greatly diminished our sample size. Given our main research objective—that is, to synthesize the breadth of literature reporting on long-term cognitive outcomes in individuals with a single mTBI—we opted for a methodological approach that would maximize the number of studies included while still balancing the need to control for limitations. In any case, the limitations posed by group homogeneity (or lack thereof) should be taken into consideration when interpreting the results.

## Conclusion

A widely cited figure in the literature suggests that only 15% of first-time concussed individuals will go on to experience persistent PCS and concomitant long-term cognitive impairment. While duly noting the limitations of our scoping review and the addressed studies, our findings suggest that this number is likely a gross underestimation at least in relation to cognitive impairment and should be carefully examined in future prospective, longitudinal studies.
