# Correction: Mild Traumatic Brain Injury (mTBI) and chronic cognitive impairment: A scoping review

**DOI:** 10.1371/journal.pone.0218423

**Published:** 2019-06-11

**Authors:** Kerry McInnes, Christopher L. Friesen, Diane E. MacKenzie, David A. Westwood, Shaun G. Boe

There is an error in reference 36. The correct reference is: Xu Z, Lv X-A, Wang J-W, Chen Z-P, Qiu H-S. Predictive value of early decreased plasma ghrelin level for three-month cognitive deterioration in patients with mild traumatic brain injury. Peptides. 2014;54:180–5. doi: https://doi.org/10.1016/j.peptides.2014.01.021.
